# Coptis Chinensis affects the function of glioma cells through the down-regulation of phosphorylation of STAT3 by reducing HDAC3

**DOI:** 10.1186/s12906-017-2029-0

**Published:** 2017-12-06

**Authors:** Jiangan Li, Lulu Ni, Bing Li, Mingdeng Wang, Zhemin Ding, Chunrong Xiong, Xiaojie Lu

**Affiliations:** 1 Department of Neurosurgery, Wuxi No.2 Hospital Affiliated to Nanjing Medical University, No. 68 Zhongshan Road, Wuxi, 214000 People’s Republic of China; 2 Department of Traditional Chinese medicin, the Affiliated Hospital of Jiangnan University ( Wuxi No.4 People’s Hospital), Wuxi, 214062 People’s Republic of China; 3 Department of Emergency medicine, Suzhou science and technology town hospital, Suzhou, 215153 People’s Republic of China; 4 Department of Neurosurgery, the Affiliated Hospital of Jiangnan, University (Wuxi No. 4 People’s Hospital), Wuxi, 214062 People’s Republic of China; 5 Research Institute of schistosomiasis control in Jiangsu Province, Wuxi, 214063 People’s Republic of China

**Keywords:** Coptis Chinensis, Glioma cells, Apoptosis, HDAC3, P-STAT3

## Abstract

**Background:**

Glioma remains the most common cause of brain cancer-related mortality. Glioma accounts for 50–60% of brain cancer. Due to their low toxicity and infrequent side effects, traditional herbs have been increasingly popular. Coptis Chinensis is commonly used in cancer treatment in combination with other Chinese Medicine herbs. However, little is known about its biological functions and mechanisms in glioma cells.

**Methods:**

In this study, the anti-glioma cell effect of Coptis Chinensis was determined using the 3-(4,5-dimethyl-2-thiazolyl)-2,5-diphenyl-2-H-tetrazolium bromide (MTT) method, plate clone test, scratch tests, flow cytometry, western blotting and a glioma xenograft tumor model.

**Results:**

The results showed that Coptis Chinensis significantly suppressed glioma cell proliferation, tumor formation, migration and tumor growth, and prolonged the survival time of glioma cell-bearing mice. The flow cytometry result showed that Coptis Chinensis induced cell cycle arrest and apoptosis in glioma cells. Western blotting showed that Coptis Chinensis down-regulated the Signal transducer and activator of transcription 3 (STAT3) phosphorylation levels and reduced the expression of Histone deacetylase 3 (HDAC3) and caspase 3.

**Conclusions:**

Coptis Chinensis can inhibit various aspects of glioma cell functions. This study provides favorable scientific evidence for the potential use of natural products such as Coptis Chinensis in the clinical treatment of patients with glioma.

## Background

Glioma is the most common primary malignant tumor in the central nervous system. Glioma has a poor prognosis and accounts for 50–60% of intracranial tumors [1]. Statistically, even for patients with low grade glioma treated with surgery combined with radiation and chemotherapy, the average survival time is only approximately 3 to 5 years, and for high grade glioma, the survival time is 1 to 2 years [2]. There is large recurrence rate, mainly because diffuse glioma tumor cells ubiquitously infiltrate into normal parenchyma. Its irregular shape makes it difficult to accurately know its location. Surgery is the first-line treatment for glioma, with radiation and chemotherapy as adjuvant therapeutic treatments which tend to produce larger adverse reactions in patients.

In recent years, with the rise of the return-to-nature boom, people have turned to nature to find means to treat diseases. Traditional Chinese Medicine (TCM) has received wide attention and research because of its relative safety and long history [3]. Qi, blood and turbid phlegm obstruct the vascular channels and collaterals in the brain, which leads to brain tumors. Hence, internal stagnation of heat toxin is a key cause of brain tumors. According to *Nei Jing·Su Wen·On True Essentials*, treatment of heat diseases requires the use of cold herbs. Therefore, herbs with a cold nature are used to clear heat and eliminate toxins in the treatment of cancer. As a basis for the therapy, herbs that clear heat and eliminate toxins can ward off pathogenic microorganisms, defend against toxins and inflammation and improve immunity. Therefore, such herbs are irreplaceable in the prevention and treatment of malignant tumors [4]. Coptis Chinensis, which removes heat and eliminates toxins, has been recorded in the essential medical classics of various dynasties. In TCM, Coptis Chinensis has the function of clearing heat, eliminating toxins, drying dampness and purging fire. *Shang Han Lun*, a famous TCM classic, has recorded 113 formulas, 12 of which contain Coptis Chinensis as an ingredient. Thus, Coptis Chinensis is considered to be one of the most frequently used herbs.

STAT protein family of transcription factors has important biological effects. Phosphorylation and acetylation of these proteins are important forms of post-translational modification that regulate the active site of many proteins [5]. The precise control of the STAT signaling pathway is essential for adaptations of the body to the environment and in the process of maintaining homeostasis, and abnormalities in this signaling pathway lead to the immune function and cell growth disorders [6–10]. Histone deacetylase inhibitor (HDACi) can inhibit histone deacetylases and regulate gene expression of tumor suppressors, thereby changing the biological characteristics of tumor. Recent studies have confirmed that VPA is one of the specific inhibitors of histone acetyltransferases (HDACs) [11], and considerable research has been performed to investigate its anti-tumor effects.

This study was guided by the theories of traditional Chinese medicine and combined in vitro and in vivo experiments with modern molecular methods. We investigated the effect of Coptis Chinensis in glioma cells and the associated biological mechanisms, and we further studied the effect of Coptis Chinensis on the relationship between STAT3 and HDAC3 to clarify whether Coptis Chinensis functioned similarly to Sodium Valproate (VPA), which down-regulated the phosphorylation of STAT3 by reducing the expression of HDAC3. This subsequently affected the function and biological characteristics of glioma cells. Our research examined the heat-clearing and detoxifying effects of Coptis Chinensis with respect to its anti-glioma functions to explore the effect and mechanism of the ‘anti-tumor’ activity and further elucidate the potential value of traditional medicine in the prevention and control of glioma with the aim of providing a basis for the clinical treatment of glioma.

## Methods

### Experimental reagents

Coptis Chinensis granules were purchased from Tianjiang Pharmaceutical Limited Company (Jiangyin, China). The granules were dissolved in 1 × PBS at 80 °C or 30 min, centrifuged at 2000 rpm for 5 min, filtered (0.22 μm), and stored at −20 °C. VPA powder dissolved in sterile DMSO at a concentration of 50 mM to provide a stock solution and stored at −80 °C was purchased from Shanghai Selleck Company. Dulbecco’s Modified Eagle’s Medium (DMEM) for culture was purchased from Gibco (Grand Island, NY, USA), fetal bovine serum (FBS) was obtained from HyClone (South Logan, USA), and trypsin was purchased from Gibco (Grand Island, NY, USA). The thiazole reagent [3-(4,5-di methyl thiazol-2-yl)-2,5-diphenyltetrazolium bromide, Thiazol blue (MTT)] was purchased from Sigma (St. Louis, MO, USA). Propidium iodide (PI) and FITC-Annexin V were purchased from BD Biosciences (San Jose, CA).

### Cell culture

The glioma cell lines, namely U251, U87, H4, LN229 and BV2 cells, were purchased from Shanghai Cell Bank of Chinese Academy of Sciences. All cell lines were cultured in DMEM (Gibco, Life Technologies, Grand Island, NY). The DMEM was supplemented with 10% FBS. The cells were maintained at 37 °C in a humidified 5% CO_2_ atmosphere.

### Microscopic observations of the effects of Coptis Chinensis granules on glioma cell morphology

To assess the effect of Coptis Chinensis on the morphology of glioma cells, the cells were examined microscopically. U251, U87, H4 and LN229 cells in logarithmic phase were seeded in a 48-well plate at a density of 5000 cells/well and maintained at 37 °C, 5% CO_2_ for 16 h. Then, the Coptis Chinensis solutions at concentrations of 0, 2.5 or 5 mg/ml were added, then maintained at 37 °C, 5% CO_2_ for 24 h, after which they were examined under the microscope and photographed.

### MTT method to determine the influence of Coptis Chinensis on the inhibition of glioma cell proliferation

To assess the effect of Coptis Chinensis on the survival and proliferation of glioma cells, MTT colorimetric analysis was used. U251, U87, H4, LN229 and BV2 cells in logarithmic phase were seeded in a 96-well plate at a density of 3000 cells/well and maintained at 37 °C, 5% CO_2_ for 16 h. Then, the different concentrations of Coptis Chinensis solutions were added. The total volume was 200 μl, 4 parallel wells in each group and were maintained at 37 °C, 5% CO_2_ for different time point. At the target times, 20 μl of the MTT reagent (Sigma) was added to each well in the 96-well plate. The plate was then incubated at 37 °C for 4 h. After removing the supernatants, 150 μl of DMSO was added, the plate was maintained at 37 °C for 15 min. The absorbance (A) at 570 nm was determined using a microplate reader. The cell inhibition was calculated as follows:

Inhibition rate (%) = [(A of negative control group – A of test group)/ A of negative control group] × 100%.

### Plate cloning experiment to test the effects of Coptis Chinensis on glioma cell colony formation efficiency

To clarify the effect of Coptis Chinensis on the tumorigenic potential of the glioma cells, a plate cloning assay was applied. U251 and U87 cells (500 for each type) in logarithmic phase were seeded in 6-well plates and cultured for 16 h. Then, Coptis Chinensis solutions at concentrations of 0, 0.625, 1.25 or 2.5 mg/ml were added to the U251 and U87 cells for 24 h, and the cells were allowed to grow for an additional 12 days at 37 °, 5% CO_2_. Twelve days later, the colonies were stained with crystal violet, and photographs of the stained colonies were taken using a digital camera and a dissecting microscope.

### Determination of the effects of Coptis Chinensis on transplanted tumors in Balb/c nude mice

Female mice at 5 weeks age and weighing 18–22 g were purchased from SLRC Shanghai and fed standard feed in Shanghai University of Traditional Chinese Medicine. U87 cells (3 × 10^6^) were suspended in medium at 50 μl and then subcutaneously injected into the right axilla of the Balb/c nude mice (*n* = 5). After the 3 days of U87 cells were injected, the mice were randomly grouped. The Coptis Chinensis groups were administered a Coptis Chinensis solution (20 or 10 mg/per mouse/day) and the blank control group was administered distilled water intragastrically for 30 days. Tumor volume was measured every 3 days with vernier calipers. The formula for the volume was as follows: V = (П/8) × a × b^2^, where “a” represents the maximum diameter of the tumor, while “b” reflects the shorter diameter perpendicular to “a”. The Balb/c nude mice with tumors over 2000 mm^3^ were killed by injecting air through the caudal vein.

### Scratch test to determine the migration ability of glioma cells

To determine the effects of Coptis Chinensis on the migration of glioma cells, a cell scratch test was used. U251 and U87 cells (6 × 10^5^)in logarithmic phase were seeded in a 6-well plate for 16 h, then cells were scratched with a scraper. The detached cells were removed with PBS. Simultaneously, Coptis Chinensis solutions at concentrations of 0, 0.3125, 0.625 or 1.25 mg/ml were added to the U251 and U87 cells. Photographs were taken using a microscope 24 h after the treatment, and the distances that the cells migrating were compared.

### Flow cytometry to test the effects of Coptis Chinensis on the glioma cell cycle

U251 and U87 cells were seeded at 5 × 10^5^ cells per plate in 10-cm plates. After 16 h, the cells were exposed to 0, 0.625, 1.25 or 2.5 mg/ml of Coptis Chinensis for 24 h and then harvested and washed with ice-cold PBS. The cells were fixed with 70% ethanol overnight at 20 °C and stained with PI for 30 min at 37 °C in the dark. The DNA content was measured using a FACSCalibur cytometer (BD Biosciences) and analyzed using FCS Express v 2.0 software. The distribution of cells at different stages of cell cycle was determined using ModFit software (Verity, Software House, Topsham, ME).

### Flow cytometry to determine the effects of Coptis Chinensis on the early apoptosis and apoptosis of glioma cells

U251 and U87 cells (1.2 × 10^5^) in logarithmic phase were seeded in a 6-well plate. Sixteen hours later, Coptis Chinensis solutions at 0, 0.625, 1.25 or 2.5 mg/ml were added to U251 and U87 cells for 24 h. After collection, the cells were incubated with Annexin V and PI. Then, the incubated cells were evaluated using a FACSCalibur cytometer (BD Biosciences) and analyzed using FlowJo software.

### Western blotting to detect the influence of HDAC3, P-STAT3 and Caspase 3 after the treatment of Coptis Chinensis

U251 and U87 cells in logarithmic phase were seeded in 10-cm culture dishes. When the cells occupied 80% of the dish, the effects of Coptis Chinensis were tested. Coptis Chinensis or VPA was added to the U251 and U87 cells. After 24 h, 100 μl of RIPA was added, and cells were fully dispersed. After centrifugation for 30 min, the supernatants were collected for protein measurement. After immersing the samples in 95 °C water for 10 min, the proteins were separated by gel electrophoresis and transferred to a membrane. The membranes were incubated overnight at 4 °C with primary antibodies and then further incubated with secondary antibodies and finally visualized.

### Statistical analysis

The statistical software SPSS version 13.0 was used for analysis. All data are presented as the mean ± standard deviation. Statistical analysis was performed using analysis of variance. Differences were judged to be statistically significant at *P* < 0.05.

## Results

### The changes in the morphology of glioma cells after treatment with Coptis Chinensis

**Fig. 1:**
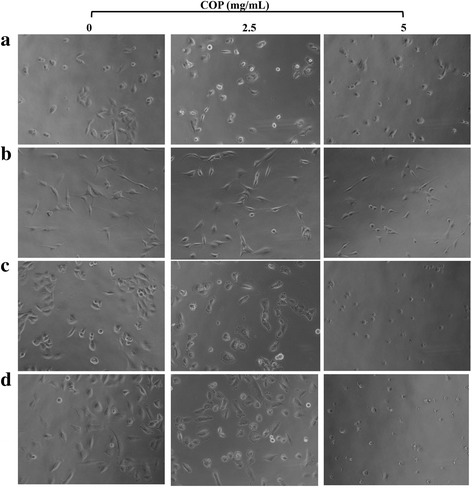
Effect of COP on proliferation of Glioma cells. U251 (**a**), U87 (**b**), H4 (**c**) and LN229 (**d**) cells were treated with COP (0, 2.5 and 5 mg/ml) for 24 h, respectively. Photographs of cell are shown

To assess the effect of Coptis Chinensis on the morphology of glioma cells, they were observed using microscopy. U251, U87, H4 and LN229 cells in logarithmic phase were treated with Coptis Chinensis at 0, 2.5 or 5 mg/ml. After 24 h, the cells were examined and photographed. We found that the morphology of the glioma cells treated with Coptis Chinensis changed significantly. Specifically, the cell membrane was observed to shrink, and the cell morphology became indistinct and circular (Fig. 1).

### Inhibition of the proliferation of glioma cells by Coptis Chinensis

**Fig. 2:**
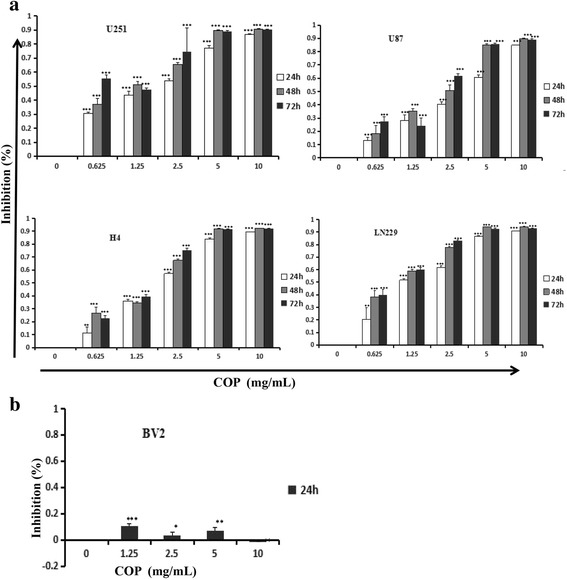
U251, U87, H4 and LN229 (**a**) were treated with COP (0, 0.625, 1.25, 2.5, 5 and 10 mg/ml) for 24, 48 and 72 h, respectively; BV2 (**b**) cells as a control cell were treated with COP (0, 1.25, 2.5, 5 and 10 mg/ml) for 24 h; cell viability was analyzed by the MTT assay. All values represent the mean ± SD from 3 independent experiments. *, *P* < 0.05; **, *P* < 0.01; ***, *P* < 0.001 compared with the control group

To study the inhibition of the growth of glioma cells by Coptis Chinensis, Coptis Chinensis at 0, 0.625, 1.25, 2.5, 5 or 10 mg/ml was applied to U251, U87, H4 and LN229 cells for 24, 48 and 72 h. The results showed that the inhibition rate for Coptis Chinensis at 0.625 mg/ml on all glioma cells was between 10% and 30%. With increases in the concentration, the inhibition was increased. In particular, the inhibition was over 30% at the higher concentrations at 24, 48 and 72 h (p<0.001). When below 5 mg/ml, the inhibition of the growth of all glioma cells was dependent on dosage and time; when at 5 mg/ml, the inhibition on all glioma cells reached the maximum (Fig. 2a). But Coptis Chinensis had no significant inhibitory effect on normal BV2 cells (Fig. 2b). These data suggested that Coptis Chinensis may be a potential inhibitor of the proliferation of glioma cells.

### Inhibition of glioma cell tumor formation by Coptis Chinensis

**Fig. 3:**
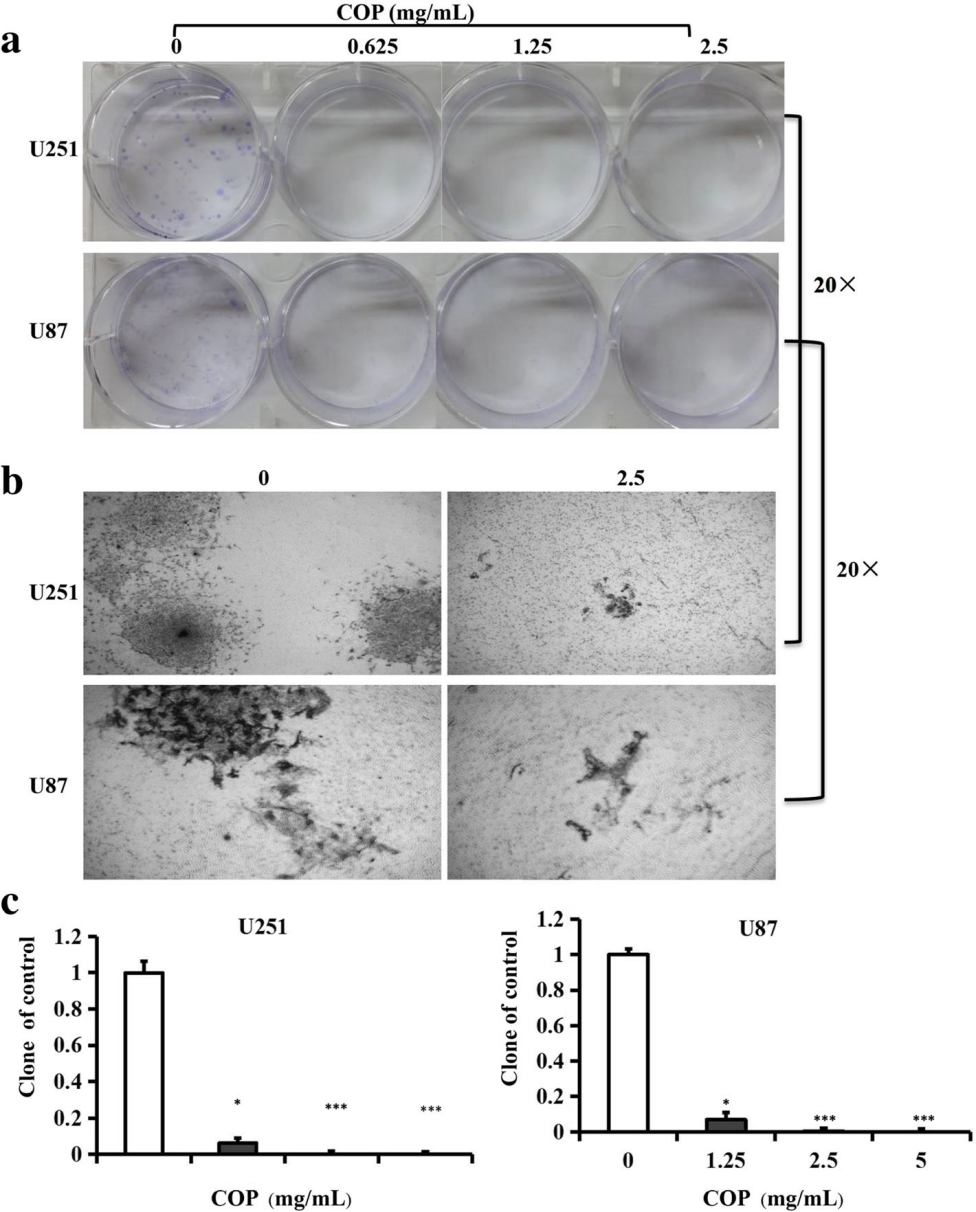
Effect of COP on colony formation of Glioma cells. U251 and U87 (**a**) cells were treated with COP (0, 0.625, 1.25 and 2.5 mg/ml) for 24 h and allowed to grow for an additional 12 days. **b** Photographs of cell culture (0 and 2.5 mg/ml) are shown. **c** Colony percentages represent the mean ± SD from 3 individual experiments. The number of colonies after treatment with vehicle control was normalized to 100%. *, *P* < 0.05; **, *P* < 0.01; ***, *P* < 0.001 compared with the control group

Previous studies indicated that Coptis Chinensis can significantly inhibit the proliferation of glioma cells. A further study on the effect of Coptis Chinensis on the tumor formation by glioma cells was performed using the plate clone assay (Fig. 3). It was found that when treated with Coptis Chinensis at 0, 0.625, 1.25 or 2.5 mg/ml, the clones of U251 and U87 cells decreased with increases in the concentration (Fig. 3a) and the photos of 2.5 mg/ml (Fig. 3b). When U251 and U87 cells were treated with 0.625 mg/ml of Coptis Chinensis, the clone formation inhibition rates were 6.25% and 6.98%, respectively (P<0.05) (Fig. 3c). Coptis Chinensis at either 1.25 or 2.5 mg/ml could completely prevent the formation of clones of the U251 and U87 cells (P<0.001) (Fig. 3a and c). These results suggest that Coptis Chinensis could greatly inhibit formation of glioma cell tumors.

### Intervention of Coptis Chinensis on the migration of glioma cells

**Fig. 4:**
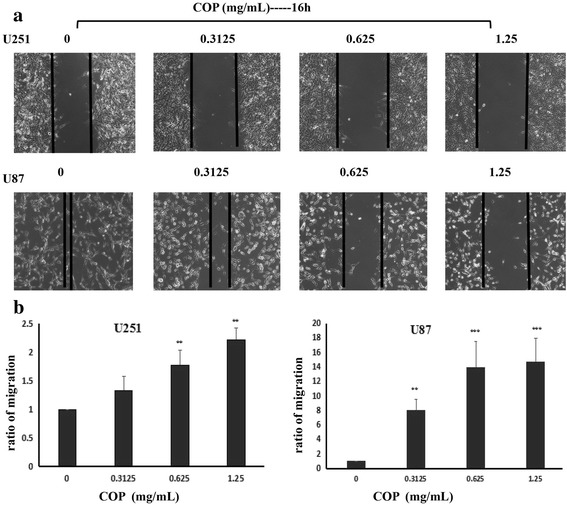
Effect of COP on Glioma cells migratory ability. **a** Scratch wound healing assays were performed, and U251 and U87 cells were treated with COP (0, 0.3125, 0.625 and 1.25 mg/ml) for 24 h. Photographs of cell culture dishes are shown. **b** Migration ratio represent the mean ± SD from 3 independent experiments. *, *P* < 0.05; **, *P* < 0.01; ***, *P* < 0.001 compared with the control group

To determine the effects of Coptis Chinensis on the migration of glioma cells, cell scratch tests were performed. According to the results of the proliferation study, when the U251 and U87 cells were treated for 24 h with Coptis Chinensis less than 1.25 mg/ml, the inhibition of glioma cell growth was relatively low. Therefore, Coptis Chinensis at 0, 0.3125, 0.625 and 1.25 mg/ml was used to treat U251 and U87 cells, and the effects of Coptis Chinensis on the migration of glioma cells were determined. As shown in the results, compared with untreated cells, Coptis Chinensis could substantially inhibit the migration of glioma cells (Fig. 4).

### Inhibition of Coptis Chinensis on the growth of glioma cells in vivo

**Fig. 5:**
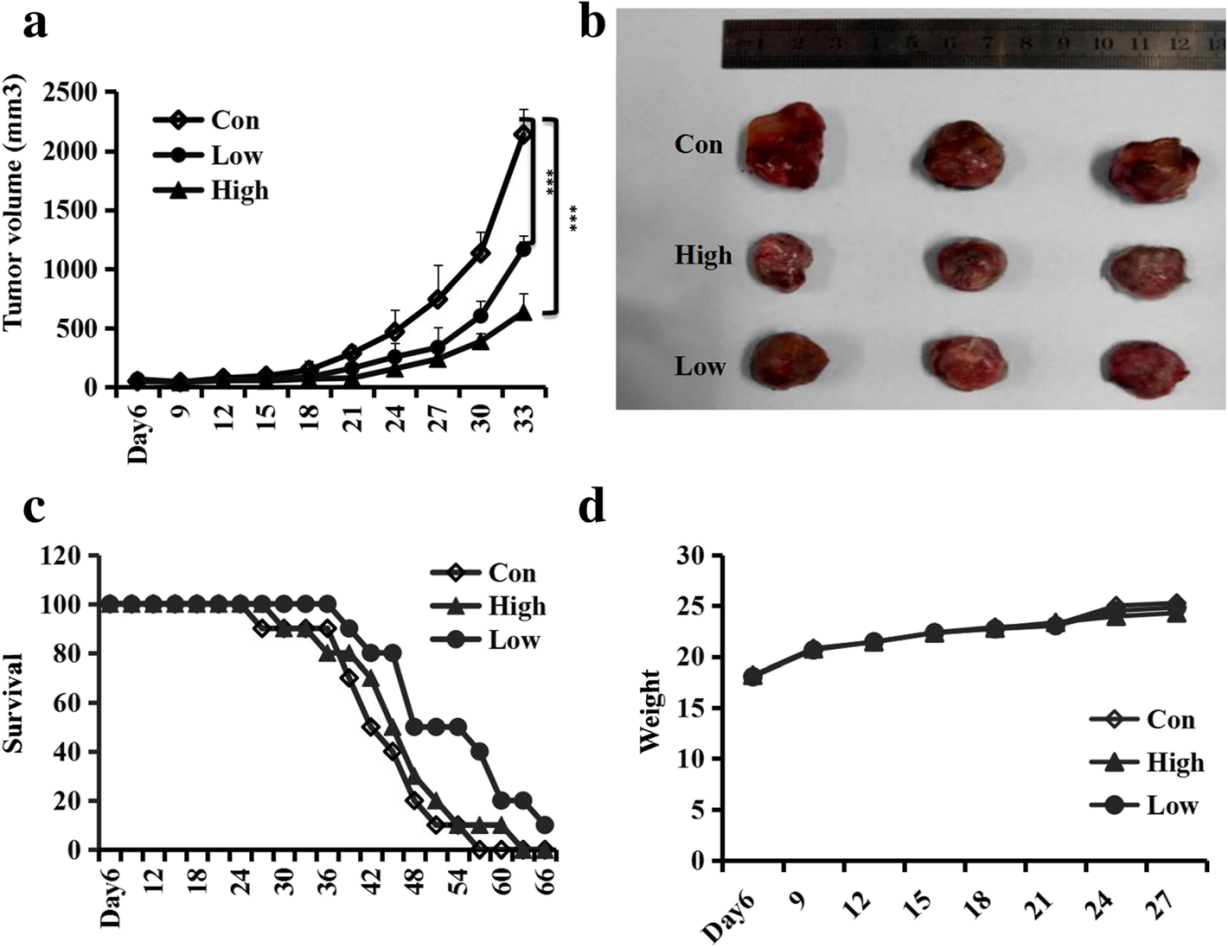
Coptis Chinensis inhibited Glioma tumor growth. U87 cells (3 × 10^6^) were subcutaneously injected into the right axilla of Balb/c nude mice. Two days after injecting, Coptis Chinensis (20 and 10 mg/per mouse/day, respectively and distilled water per mouse was administered intragastrically. **a**, **b** Tumor volume and tumor photos at different time and various concentrations points. **c**, **d** Survival and weight at different days. Data are presented as mean ± SD from three independent experiments. *, *P* < 0.05; **, *P* < 0.01; ***, *P* < 0.001 compared with the control group

Since Coptis Chinensis could inhibit the formation of glioma cell tumors, we investigated whether Coptis Chinensis could inhibit the tumor growth of glioma cells in vivo. For this purpose, Balb/c nude mice were selected for study. U87 cells were injected into right axilla of Balb/c nude mice. Coptis Chinensis at doses of 20 or 10 mg/per mouse/day or distilled water was administered intragastrically for 1 month at intervals of 1 day. Compared with the distilled water, Coptis Chinensis clearly inhibited the growth of glioma cells in vivo (Fig. 5a and b). In addition, it was found that the weight of Balb/c nude mice remained unchanged (Fig. 5d). The survival times for the groups treated with Coptis Chinensis at low and high doses and the distilled water group were 66, 60 and 54 days, respectively (Fig. 5c). These results suggested that Coptis Chinensis can markedly prolong the lifetime of mice with glioma tumors (P<0.05).

### Coptis Chinensis induces G2/M arrest in glioma cells

**Fig. 6:**
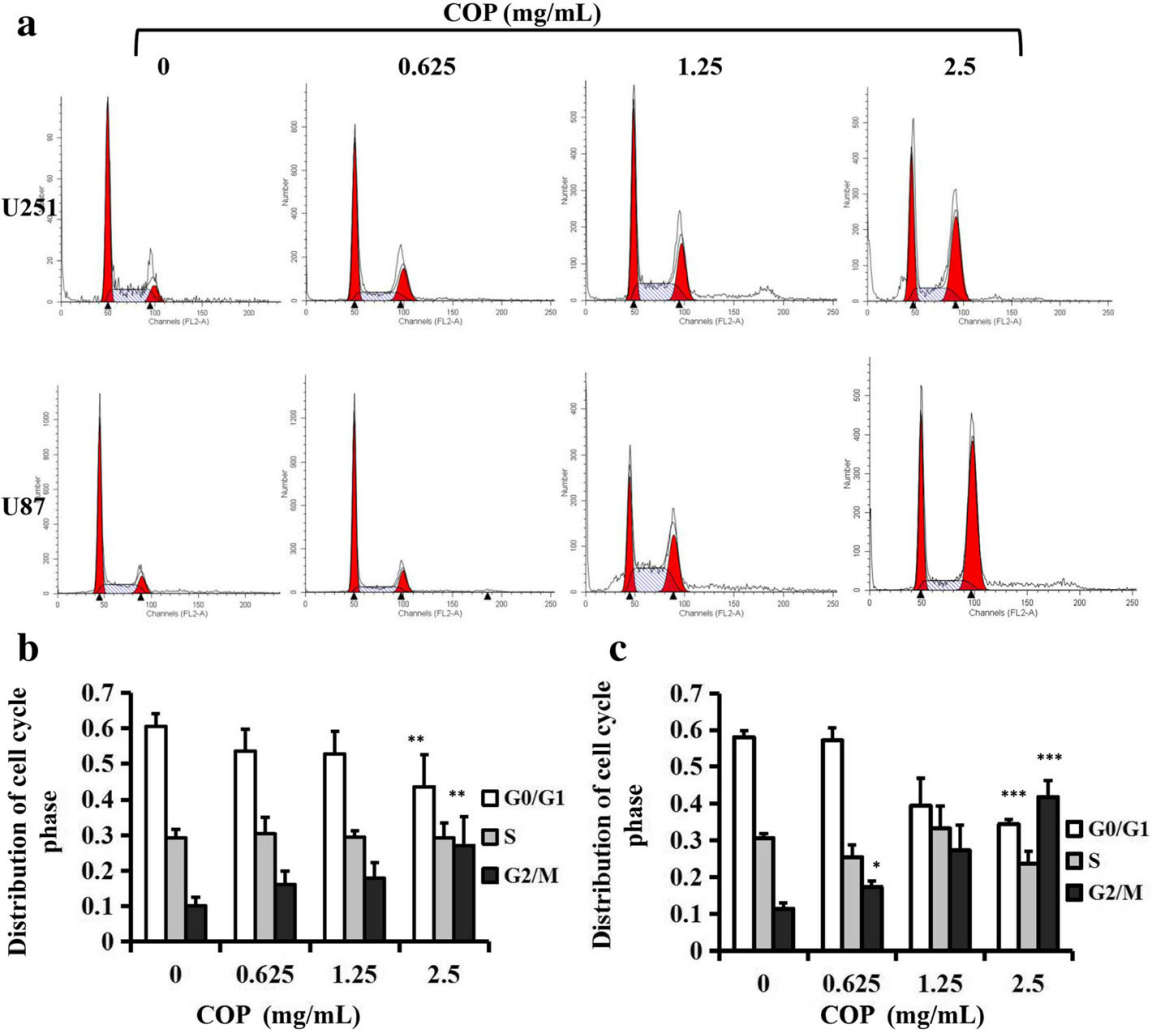
COP induces G2/M phase arrest U251 and U87 cells. **a** Flow cytometry showed U251 and U87 cells were blocked at G2/M phase. **b** U251 was arrested in G2/M phase. **c** U87 was arrested in G2/M phase. *, *P* < 0.05; **, *P* < 0.01; ***, *P* < 0.001 compared with the control group

To determine the molecular mechanisms for the inhibitory effects of Coptis Chinensis on cell proliferation, tumorigenicity and tumor growth, U251 and U87 cells were treated with various concentrations of Coptis Chinensis and the cell cycle was analyzed by flow cytometry (Fig. 6a). We observed that at 0, 0.625, 1.25 or 2.5 mg/ml of Coptis Chinensis, 10.08%, 16.06%, 17.82% and 27.11% of the U251 cells (Fig. 6b) and 11.41%, 17.39%, 27.32% and 41.81% of the U87 cells, respectively, were in G2/M phase (Fig. 6c). The proportions of the cells in G2/M phase were significantly higher after 2.5 mg/ml Coptis Chinensis treatment (Fig. 6a–c).

### Effect of Coptis Chinensis on the induction of apoptosis of glioma cells

**Fig. 7:**
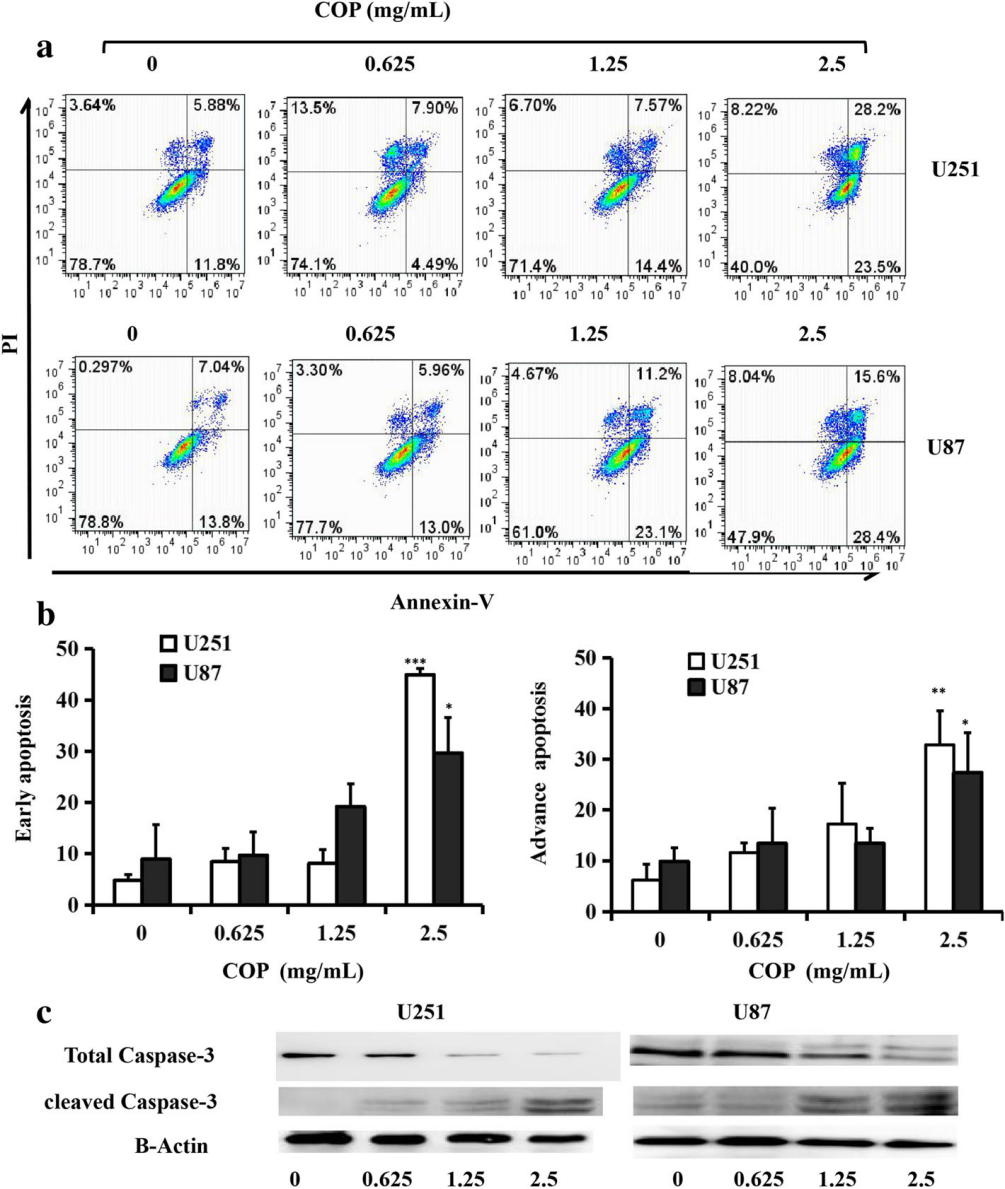
Effect of COP on cell apoptosis. **a** U251 and U87 cells were treated with COP at different concentrations (0, 0.625, 1.25 and 2.5 mg/ml) for 24 h, then stained with Annexin V-fluorescein isothiocyanate (FITC)/PI. **b** Quantitative analysis of dose-dependent alteration of COP on apoptosis. **c** U251 and U87 cells were treated with COP at different for 24 h, the expression of Caspase 3 were down-regulated and cleaved Caspase 3 were increased. Data are presented as mean ± SD from three independent experiments. *, *P* < 0.05; **, *P* < 0.01; ***, *P* < 0.001 compared with the control group

Since Coptis Chinensis markedly inhibited the growth of glioma cells, the effect of Coptis Chinensis on apoptosis was further tested. U251 and U87 cells were treated with Coptis Chinensis at various concentrations and then stained with Annexin V-fluorescein isothiocyanate (FITC)/PI. The results suggested that Coptis Chinensis can clearly induce the early stage (Annexin V-FITC^+^/PI^−^) and advanced stage (Annexin V-FITC/PI^+^) of apoptosis (Fig. 7a). U251 and U87 cells were treated with Coptis Chinensis at 0, 0.625, 1.25 and 2.5 mg/ml. The early apoptosis rates in the U251 cells were 4.75, 8.42, 8.14 and 44.9%, whereas those of the U87 cells were 8.93, 9.66, 19.2 and 29.7%, respectively and the advanced apoptosis rates in the U251 cells were 6.126, 11.55, 17.24 and 32.86%, whereas those in the U87 cells were 9.84, 13.4, 13.42 and 27.39%, respectively (Fig. 7b). The early and advanced apoptosis rates in the U251 and U87 cells treated with Coptis Chinensis increased with increasing concentrations (P<0.05) (Fig. 7a and b). The expression levels of protein associated with apoptosis changed under the treatment with Coptis Chinensis, with the expression of total Caspase 3 reducing and the expression of cleavage caspase 3 (active form of caspase-3) increasing (Fig. 7c).

The findings indicated that Coptis Chinensis can induce the apoptosis of glioma cells.

### Effect of Coptis Chinensis on the influence of HDAC3, P-STAT3 and Caspase 3 in glioma cells

**Fig. 8:**
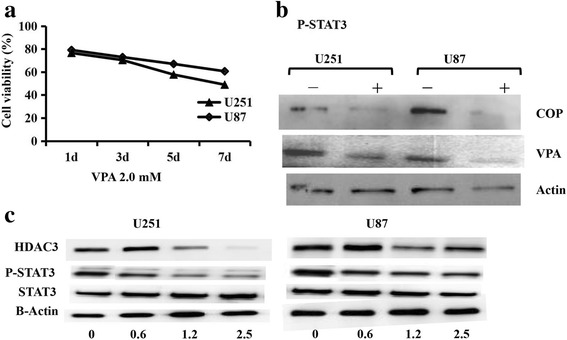
COP induced protein expression in U251 and U87 cells. U251 and U87 (**a**) cells were treated with 2.0 mM VPA for 1, 3, 5 and 7 d, respectively, cell viability was analyzed by the MTT assay. **b** showed the VPA 2.0 mM and COP 5 mg/ml reduced the expression of STAT3 phosphorylation. **c** U251 and U87 cells were treated with COP for 24 h, the expression of HDAC3 and STAT3 phosphorvlation were down-regulated

Preliminary experiments showed that Coptis Chinensis inhibited cell proliferation and induced cell apoptosis. In addition, we found that VPA also had a certain inhibitory effect on the glioma cells: 2.0 mM VPA inhibited the growth of the U251 and U87 cells by 20–30%, and the effect was dependent on time (Fig. 8a). Western blotting was then applied to determine the molecular mechanisms. U251 and U87 cells were treated for 24 h with Coptis Chinensis at 0, 0.625, 1.25 or 2.5 mg/ml (for testing P-STAT3 and HDAC3), or with 2.0 mM VPA and 5 mg/ml Coptis Chinensis(for testing P-STAT3). The proteins were then separated and used for protein analysis. The protein expression of HDAC3 and P-STAT3 was examined using western blotting. The results showed that 2.0 mM VPA or 5 mg/ml Coptis Chinensis could reduce the expression of P-STAT3 (Fig. 8b), and Coptis Chinensis down-regulated HDAC3 and P-STAT3 in glioma cells, but it did not reduce the expression of STAT3 (Fig. 8c). In conclusion, we could infer that Coptis Chinensis down-regulated the phosphorylated STAT3 level by reducing the expression of HDAC3 protein, which then affected the function of glioma cells and their biological characteristics.

## Discussion

Glioma is the most common primary malignant tumor in the central nervous system. This type of tumor accounts for 50–60% of intracranial tumors and has a poor prognosis [1]. Statistically, even for patients with low grade glioma treated with surgery combined with radiation and chemotherapy, the average survival time is only approximately 3 to 5 years, whereas the survival for patients with high grade gliomas is 1 to 2 years [2]. Traditional Chinese medicine (TCM) is a valuable natural treasure for the prevention and treatment of diseases in humans. TCM have the characteristics of multiple targets and multiple forms of intervention against tumors. Compared with surgery, radiation and chemotherapy treatment, TCM has unique advantages and great potential. In the treatment of patients with glioma, we aimed to find an optimal balance between the patients’ survival and quality of life and the adverse responses to TCM. In recent years, our country has carried out extensive and effective explorations on the compounds, extracts and active ingredients of TCM, and valuable experience and experimental data have been acquired. In this context, the heat-clearing and detoxifying effects of Coptis Chinensis in the treatment of glioma may have considerable value. Our experimental results showed that Coptis Chinensis could significantly inhibit the proliferation and migration of glioma cells and promote their apoptosis.

Coptis Chinensis has been used as an herb for clearing heat and eliminating toxins for centuries. Modern pharmacological studies found that Coptis Chinensis can defend against inflammation, ward off bacteria, decrease blood pressure and blood glucose and resist oxidation. Coptis Chinensis and its main alkaloid can be seen as a potential candidate for new treatments for Parkinson’s disease [12]. Cui reported that a Coptis Chinensis polysaccharide has the potentially therapeutic effect of suppressing the absorption of high blood glucose and glucose, thereby improving the adverse reactions of glucose tolerance [13]. The extract of Coptis Chinensis powder can be used as a form of immune enhancer that has certain effects on a weakened immune system when it is used as an anti-inflammatory drug, auxiliary material and supplementary treatment [14]. In addition, Coptis Chinensis can inhibit the hyperplasia of gastritis, gastric ulcer and gastric cancer [15], colon cancer [16, 17], and even breast cancer [18, 19]. However, little is known about the biological effects of Coptis Chinensis on glioma.

HDACi can inhibit the histone deacetylase and regulate the gene expression of tumor suppressors, which can change the biological characteristics of tumors. HDACi inhibit tumor cells by regulating tumor cell apoptosis, cell cycle, and gene expression. Recently, various HDACi have been investigated in clinical trials and have achieved some success in cancer treatment, autoimmune disease and immune rejection [20–22]. Recent studies have confirmed that VPA is one of the specific inhibitors of HDACs [11], and considerable research has been carried out on its anti-tumor effect. The STAT protein family of transcription factors has important biological effects. The precise control of STAT signaling pathway is critical to allow the body to adapt to the environment and in the process of maintaining homeostasis, and abnormalities in this signaling pathway lead to disorders of the immune function and cell growth [6–10]. Phosphorylation and acetylation are key forms of post-translational modification that regulate the active sites of many proteins [5]. Some reports have demonstrated that VPA plays an important role in regulating the function of STAT3 by reducing the STAT3 phosphorylation level in biological processes [23–25]. Unlike the transient STAT3 phosphorylation in normal cells, sustained activation of STAT3 was observed in 70% of blood and solid tumors and showed the strong dependence, which makes it an ideal drug target [26, 27].

In this study, human glioma cell lines were selected as the model and Coptis Chinensis granules as the treatment. The effect of Coptis Chinensis on the proliferation, apoptosis, migration and invasion of glioma cells was explored. MTT analysis was used to determine the survival rate of glioma cells. It was found that Coptis Chinensis could substantially inhibit the survival of glioma cells. Next, the morphological changes in the glioma cells treated with Coptis Chinensis were observed using microscopy. The plate clone assay was used to test the tumor formation ability of glioma cells. The scratch assay tested the influence of treatment with Coptis Chinensis on the migration of glioma cells. Glioma tumor transplantation in a nude mouse model was used to investigate the effect of intervention with Coptis Chinensis on glioma growth in vivo. Flow cytometry was used to evaluate the ability of Coptis Chinensis to interfere with the glioma cell cycle and apoptosis. Finally, we determined the HDAC3 protein, STAT3 phosphorylation and the core apoptosis protein Caspase 3 level in glioma cells treated with Coptis Chinensis using western blot to explore the effect of Coptis Chinensis intervention on the biological mechanisms in glioma cells. Some reports have demonstrated the relationship of STAT3 and HDAC3 in cells [28]. In this study, we found that Coptis Chinensis functioned similarly to VPA to down-regulate STAT3 phosphorylation level by reducing the expression of HDAC3, then affected the function and biological characteristics of glioma cells.

## Conclusion

In conclusion, our research investigated the heat-clearing and detoxifying effect of Coptis Chinensis as an anti-glioma agent and explored the effect and mechanism of the ‘anti-tumor’ effects. In addition, we extended the information on the potential value of traditional medicine in the prevention and control of glioma, provided a basis for the clinical treatment of glioma, and showed that Coptis Chinensis is likely to be a valuable natural product in the clinical treatment of patients with glioma.

## Abbreviations

AV-FITC: Annexin V-fluorescein isothiocyanate
HDAC3: Histone deacetylase 3
HDACi: Histone deacetylase inhibitor
HDACs: histone acetyltransferases
MTT: 3-(4,5-dimethyl-2-thiazolyl)-2,5-diphenyl-2-H-tetrazolium bromide
STAT3: Signal transducer and activator of transcription 3
TCM: Traditional Chinese Medicine
VPA: Sodium Valproate
